# Estimation of genetic parameters for semen traits in Egyptian buffalo bulls

**DOI:** 10.1007/s11250-023-03680-7

**Published:** 2023-07-10

**Authors:** Mohamed M. I. Salem, Amin M.S. Amin, Ayman F. Ashour, Ayman G. EL Nagar

**Affiliations:** 1 Department of Animal and Fish Production, Faculty of Agriculture, University of Alexandria, Alexandria, 21545 Egypt; 2 Animal Production Research Institute, Agricultural Research Center, Dokki, Giza, 12619 Egypt; 3 Department of Animal Production, Faculty of Agriculture at Moshtohor, Benha University, Benha, 13736 Egypt

**Keywords:** Bayesian analysis, Egyptian buffalo, Genetic correlation, Heritability, Repeatability

## Abstract

This study was conducted to characterize semen traits (ejaculate volume (VOL), mass motility (MM), sperm livability (LS), percentage of abnormal sperms (AS), and sperm concentration (CONC)) of Egyptian buffalo bulls and evaluate the importance of some nongenetic factors (year (YC) and season (SC) of semen collection and age of bull genetically and environmentally at collection (ABC)) affecting the investigated traits. A total of 7761 normal semen ejaculates were collected from 26 bulls from 2009 to 2019. Single-trait and bivariate repeatability animal models using Bayesian methods were used to estimate variance components, heritability, repeatability, and genetic correlations among the investigated semen traits. YC and ABC exerted significant effects on most semen traits, whereas SC exerted no significant effect on all the investigated semen traits. Heritability estimates were 0.08, 0.52, 0.51, 0.04, and 0.49 for VOL, MM, LS, AS, and CONC, respectively. Repeatability estimates were 0.14, 0.82, 0.79, 0.06, and 0.78 for VOL, MM, LS, AS, and CONC, respectively. The genetic correlations between MM and each of LS and CONC were highly significant (0.99 ± 0.01 and 0.95 ± 0.14, respectively), and that between LS and CONC was also highly significant (0.92 ± 0.20). The high heritability estimates for MM, LS, and CONC combined with the favorable high significant genetic correlations between these traits indicated that direct selection for MM may be an effective method to enhance semen quality in Egyptian buffalo bulls and consequently improve fertility.

## Introduction

In dairy cattle, fertility is considered one of the most essential economic characteristics (Druet et al., 2009). In several countries, dairy cows’ fertility has been extensively examined in various cattle breeds for breeding purposes, whereas bull fertility has received limited attention (Weigel et al., 2006; Yin et al., 2019). Nevertheless, male fertility in cattle can be assessed either directly on the semen or indirectly via the females. The genetic makeup of bulls and environmental factors such as season (ambient temperature), age of bulls, bull handlers, and the frequency of semen collection all influence semen traits (Stälhammar et al., 1989; Fuerst-Waltl et al., 2006). Moreover, a better understanding of these factors may enable the industry to improve artificial insemination (AI) in bull management to improve semen production (Mathevon et al., 1998). Furthermore, semen quality parameters are considered to be the most essential indications of bull fertility (Kumar et al., 2014; Singh et al., 2017), because low semen quality is a primary cause of female conception failure that has an impact on farm animals’ profitability as the use of bulls with unknown fertility in service, which reduces overall field fertility, is one of the key constraints to maximizing buffalo production (Oliveira et al., 2013).

In dairy cattle and buffalo, AI is the most effective strategy for genetic improvement. AI aims to improve productive and reproductive traits by introducing daughters from superior breeding bulls into their herds (Mathevon et al., 1998; Kattab et al., 2022). Moreover, several studies have reported positive moderate-to-high genetic and phenotypic correlations between semen traits and nonreturn rate of dairy bulls (Morrell et al., 2018; Bhave et al., 2022; Gebreyesus et al., 2021). For instance, Berry and Kearney (2011) reported a positive (0.52) genetic correlation between male semen traits and female pregnancy rate, implying that selection for semen quality will lead to a parallel improvement in the female population’s pregnancy rate of dairy cows. In Egypt, the conception rate of buffalo cows is low and directly related to bulls’ semen quality. Hence, determining the genetic factors for semen traits could provide valuable information that can be used to increase conception rates in the Egyptian buffalo. In addition, the heritability and genetic correlations among different sperm quality traits can be used to predict how these traits will respond to genetic selection. Moderate to high heritabilities for semen traits in dairy bulls have been reported in several studies (Mathevon et al., 1998; Druet et al., 2009; Khattab et al. 2015; Berry et al., 2019; Khattab et al., 2022). Previous studies have also reported favorable genetic correlations between semen traits in dairy bulls, implying that an improvement in any trait will be accompanied by an improvement in the other traits as a correlated response due to selection (Berry et al., 2014; Olsen et al., 2020). However, there are limited studies on the genetic and environmental evaluation of semen traits in buffalo. Therefore, the objectives of the present study were to (1) evaluate the importance of some nongenetic factors (i.e., year and season of semen collection, age of the buffalo bull at semen collection) on semen traits (ejaculate volume, sperm mass motility, live sperm percentage, abnormal sperm percentage, and sperm concentration) and (2) estimate variance components, heritability, repeatability, and genetic and phenotypic correlations for the examined semen traits. The achievement of these objectives will allow for better understanding of the genetic basis of semen traits in the Egyptian buffalo, as well as contributing to their genetic improvement and evaluating the possibility of incorporating routine semen examination records into the genetic evaluation of fertility.

## Material and methods

### Animals and investigated traits

A total of 7932 semen ejaculates were collected throughout 9 consecutive years from 2009 to 2018 from 26 Egyptian buffalo bulls weighing 350–400 kg live body weight and raised in the International Livestock Management Training Center (IMTC) at Sakha, Kafr El-Sheikh Governorate, belonging to the Animal Production Research Institute, Ministry of Agriculture, Dokki, Cairo, Egypt. According to NRC (2001), the bulls were selected for the first time depending on scrotal size according to methods described by Paint et al. (2003), whereas bulls with as between 18 and 24 months and crotal size more 19 cm were used for insemination. The bulls were daily fed on a ration consisting of 4 kg concentrate feed mixture, 3 kg clover hay, and 4 kg rice straw. The average temperature, relative humidity, and THI in summer at Sakha, Kufr El-Sheikh Governorate, were 28.3, 65.8, and 78.2, respectively, while the corresponding values in winter were 16.1, 75, and 60.5, respectively. Each bull’s semen was collected twice a week at 8 AM through an artificial vagina (IMV, France), maintained at 42–45°C, and immediately transferred in a water bath (37°C) to the IMTC laboratory for further semen evaluation. The investigated semen traits were ejaculating volume (VOL; mL), mass motility (MM; %), live sperm (LS; %), abnormal sperm (AS; %), and sperm concentration (CONC; 10^9^ spermatozoa/mL). Using a graduated glass tube, the ejaculate volume was measured directly in milliliters to the nearest 0.1 ml. The percentage of spermatozoa wave motion in a drop of semen deposited on a glass slide was used to calculate semen mass motility (%). The abnormal sperm (%) were measured according to the procedure adopted by Blom (1983) and Barbas and Mascarenhas (2009). The sperm concentration (10^9^ spermatozoa/mL) in each ejaculate was determined using a Neubauer hemocytometer. Percentage of livability, motility of spermatozoa, then sperm count was estimated by using a warm microscope stage in post-diluted, post-equilibrated and post-thawed semen adjusted at 37°C. Research microscope with high power magnification (×400) and warmed stage (37°C) was used to estimate the percentage of progressive sperm motility (Amman and Hammerstedt, 1980). The livability percentage of sperm cells was assessed by using eosin and nigrosine combination stain (Hackett and Macpherson, 1965). Dead sperm (stained ones) and live sperm (unstained ones) were calculated at field of 200 sperm cells. Percentage of intact acrosome was conducted as showmen by Watson (1975). Sperm morphology estimation has been described through a bright-field microscope by noticing stained semen ejaculates.

### Statistical analysis

An analysis using the GLM procedure of SAS (SAS, 2014) was conducted to evaluate the significance of the nongenetic environmental effects of season of semen collection, year of semen collection, and age of the buffalo bull by fitting the following linear model:

$${Y}_{ijkl}=\mu +{S}_i+{T}_j+{A}_k+{e}_{ijkl},$$

where *Y*_*ijkl*_=the observation of the examined trait (ejaculate volume, mass motility, live sperm, abnormal sperm, or sperm concentration); *μ* = a trait-specific underpinning constant; *S*_*i*_ = the fixed effect of the *i*th season of semen collection (four levels; spring, summer, autumn, and winter); *T*_*j*_ = the fixed effect of the *j*th year of semen collection (five levels; 2009–2010, 2011–2012, 2013–2014, 2015–2016, and 2017–2019); *A*_*k*_ = the fixed effect of the *k*th age of the bull at semen collection (five levels; ≤24, 25–36, 37–60, 61–90, and >96 months), and *e*_*ijkl*_ is the residual of the model.

A Bayesian approach was used to estimate variance components and population genetic parameters for the investigated semen traits using the THRGIBBS1F90 software (Tsuruta & Misztal, 2006) which was based on sample statistics from marginal posterior distribution produced using a Gibbs sampling algorithm. The Gibbs sampling algorithm consisted of 500,000 iterations, discarding the first 50,000. Further, one sample in each 40 was saved, and features of interest of the marginal posterior distributions were obtained using the POSTGIBBSF90 software (Tsuruta & Misztal, 2006). The single-trait repeatability animal model was used to estimate heritability and repeatability as follows:

$$\boldsymbol{Y}=X\boldsymbol{b}+Z\boldsymbol{d}+W\boldsymbol{a}+L\boldsymbol{pe}+\boldsymbol{e}\ .$$

Moreover, a set of bivariate repeatability animal models was used to estimate the genetic and phenotypic correlations between each pair of semen traits, as follows:

$$\left[\begin{array}{c}{y}_1\\ {}{y}_2\end{array}\right]=\left[\begin{array}{cc}{x}_1& 0\\ {}0& {x}_2\end{array}\right]\left[\begin{array}{c}{b}_1\\ {}{b}_2\end{array}\right]+\left[\begin{array}{cc}{z}_1& 0\\ {}0& {z}_2\end{array}\right]\left[\begin{array}{c}{d}_1\\ {}{d}_2\end{array}\right]+\left[\begin{array}{cc}{w}_1& 0\\ {}0& {w}_2\end{array}\right]\left[\begin{array}{c}{a}_1\\ {}{a}_2\end{array}\right]+\left[\begin{array}{cc}{L}_1& 0\\ {}0& {L}_2\end{array}\right]\left[\begin{array}{c}{pe}_1\\ {}{pe}_2\end{array}\right]+\left[\begin{array}{c}{e}_1\\ {}{e}_2\end{array}\right],$$

where *Y* = vector of the observed semen trait; ***b*** = vector of the fixed effects of season of semen collection, year of semen collection, and the age of buffalo bulls with a design matrix *X*; ***d*** = vector of test-day effects with design matrix *Z*; ***a*** = vector of random effects of the additive genetic effect with a design matrix *W*; ***pe*** = vector of non-additive permanent environmental effects with a design matrix *L*; and ***e*** = vector of random residual effects. Residuals were normally distributed with mean 0 and variance *I*_*n*_*σ*^2^_*e*_, where *I*_*n*_ is the identity matrix with the number of records as its order, and *σ*^2^_*e*_ is the residual variance. The vector of additive (animal) genetic effects was assumed to have a normal distribution with mean 0 and variance *Aσ*^2^_*a*_, where *A* is the numerator relationship matrix based on the pedigree, and *σ*^2^_*a*_ is the additive genetic variance. The vector of the permanent environmental effects was assumed to be normally distributed with mean 0 and variance *I*_*c*_*σ*^2^_*pe*_, where *I*_*c*_ is the identity matrix with the number of buffalo bulls as its order, and *σ*^2^_*pe*_ is the permanent environmental variance. Heritability (*h*^2^) was calculated as the ratio of genetic variation due to the additive genetic variance to the total phenotypic variance (${h}^2=\frac{{\sigma^2}_a}{{\sigma^2}_p}$). Repeatability (*r*) was calculated by dividing the additive genetic variance plus permanent environmental variance on the total phenotypic variance ($r=\frac{{\sigma^2}_{a+}{\sigma^2}_{pe}}{{\sigma^2}_p}$).

## Results and discussion

### Actual mean and variation of semen traits in Egyptian buffalo bulls

Descriptive statistics of the investigated traits are presented in Table 1. The overall mean values for VOL, MM, LS, AS, and CONC were 3.89 mL, 62.37%, 60.64%, 3.94%, and 0.67 × 10^9^ sperm/mL, respectively. These values were within the range reported by previous studies for dairy and buffalo bulls (Khattab et al., 2015; Khattab et al., 2022). In Egyptian buffalo bulls, Mahmoud et al. (2013) reported the overall mean values for VOL, MM, and LS as 2.9 mL, 70.9%, and 65.8%, respectively. Khattab et al. (2015) reported the actual mean values for VOL, MM, and LS as 3.26 mL, 58.89%, and 65.96%, respectively. Similarly, Gabr and El Basuini (2018) reported mean values for VOL, LS, AS, and CONC as 2.6 mL, 67.3%, 18.4%, and 526.28 × 10^6^ sperm/mL, respectively. Furthermore, Rushdi et al. (2017) reported values of 66.20%, 70.58%, and 15.15% for MM, LS, and AS, respectively. Differences in genetic makeup, reproductive and health status of bulls, age of bulls, frequency of collection, collection teamwork, nutrition, season and year of collection, and management may explain the differences in mean values for semen traits observed in this study and those reported by different researchers working on different breeds of dairy or buffalo bulls (Khattab et al., 2015; Khattab et al., 2022). The coefficient of variation (CV%) for semen traits ranged from 25.17 to 46.57%. The highest CV% for ejaculate volume in the present study (46.57%) represents the wide variation in semen volume between bulls. These estimates were consistent with those reported by Khattab et al. (2015) who reported a CV% range of 21.86–38.61% for semen traits in Egyptian buffalo bulls.

**Table 1:** Actual mean, standard deviation (SD), coefficient of variation (CV), minimum (min), and maximum (max) values for semen traits in Egyptian buffalo bulls

Trait*	Mean	SD	CV%	Min	Max
Ejaculate volume (mL)	3.89	1.81	46.57	0.50	11.50
Mass motility (%)	62.37	15.85	25.41	12.00	94.00
Live sperm (%)	60.64	15.26	25.17	10.00	87.00
Abnormal sperm (%)	3.94	1.72	43.53	1.00	9.00
Concentration^†^	0.67	0.16	24.49	0.20	0.95

*Total number of records is 7761

^†^Sperm concentration (10^9^ spermatozoa/mL)

### Nongenetic factors affecting semen traits

Table 2 shows the least square mean values of semen traits as affected by year of semen collection (YC), season of semen collection (SC), and the bull’s age at collection (ABC). YC and ABC were found to exert significant effects on most semen traits, indicating the need to focus on the importance of adjusting the semen data for these factors when estimating variance components and predicting breeding values. YC exerted highly significant (*P* = 0.0001) effects on MM, LS, and CONC but not on VOL and AS. However, ABC exerted significant (*P* = 0.0001) effects on VOL, MM, LS, and CONC but not on AS, considering that AS remained significantly unaffected by any one of the nongenetic examined factors. The season of semen collection exerted no significant effect on all the examined semen traits. It is possible that the relevant effect of year of collection on most semen characteristics is due to differences in nutrition, climate, and management procedures across years (Khattab et al., 2015). Moreover, Taylor et al. (1985) reported that the month–year subclass induced a substantial effect on semen characteristics in Holstein bulls, which could be related to environmental trends. As the age of bulls at semen collection advanced, the ejaculate volume increased, whereas the mass motility and sperm concentration decreased significantly (Table 2). This implies that the ejaculate volume was significantly higher in old and adult bulls than in young buffalo bulls. This result agreed with that reported by Khattab et al. (2015), who mentioned that the age of bulls might be one of the most important factors impacting sperm characteristics (volume, livability, and motility). Al-Kanaan et al. (2015) also found that the semen ejaculate volume in Holstein Friesian bulls increased as the bull’s age advanced, and the VOL values were 4.11, 4.52, 5.69, and 7.16 mL for bulls aged ≤12, 12–17, 18–47 and ≥48 months, respectively.

**Table 2:** Least square mean values for nongenetic factors affecting semen traits (Total number of records is 7761) in Egyptian buffalo bulls

Factor	*N*	Ejaculate volume (mL)	Mass motility (%)	Live sperm (%)	Abnormal sperm (%)	Concentration^†^
Year of semen collection						
2009–2010	1586	3.85	60.26^c^	57.20^c^	3.85	0.67^c^
2011–2012	2961	3.98	59.02^c^	57.48^c^	3.96	0.64^d^
2013–2014	2231	3.92	66.71^b^	62.25^b^	3.97	0.71^b^
2015–2016	1008	3.83	72.67^a^	70.80^a^	3.94	0.77^a^
2017–2018	146	3.79	71.41^a^	70.60^a^	3.97	0.76^a^
*P* value		0.0540	0.0001	0.0001	0.9512	0.0001
Season of semen collection						
Spring	2051	3.87	63.59	61.65	3.92	0.685
Summer	2003	3.92	63.54	61.62	3.94	0.685
Autumn	1951	3.99	63.26	61.38	3.97	0.682
Winter	1957	3.88	63.12	61.26	3.92	0.678
*P* value		0.348	0.065	0.065	0.713	0.062
Age of bull						
≤24 months	154	3.65^b^	66.94^a^	63.35^a^	3.81	0.75^a^
25–36 months	302	3.72^b^	65.83^ab^	62.29^a^	3.66	0.76^a^
37–60 months	1311	3.79^b^	60.03^d^	57.51^b^	3.90	0.66^c^
61–90 months	2962	3.85^ab^	63.62^c^	61.75^a^	3.95	0.69^b^
>96 months	3233	4.05^a^	64.12^bc^	62.67^a^	3.97	0.68^b^
*P* value		0.0001	0.0001	0.0001	0.2107	0.0001

Mean values superscripted with different letters within each factor and column are significantly different

^†^Sperm concentration (10^9^ spermatozoa/mL)

### Variance components and genetic parameters

The estimates of variance components, heritability, and repeatability for semen traits are given in Table 3. For all the investigated traits, the estimates of the additive genetic variance were relatively higher than those of the permanent environmental variances and the variances due to test day as well. The heritability estimates were 0.08, 0.52, 0.51, 0.04, and 0.49 for VOL, MM, LS, AS, and CONC, respectively. The repeatability estimates were 0.14, 0.82, 0.79, 0.06, and 0.78 for VOL, MM, LS, AS, and CONC, respectively. Sperm mass motility, percentage of live sperms, and sperm concentration had higher heritability estimates than the other traits. Therefore, selection for MM, LS, and CONC in this herd of Egyptian buffalo bulls may result in rapid genetic improvement in semen characteristics and fertility. Considering that the estimates of genetic correlation between the three traits were favorably positive, ranging from 0.92 and 0.99 (Table 4), the selection for MM as an easy trait for estimation might be accompanied by the desired improvement in both LS and CONC traits. The estimates in this study were higher than those reported by Khattab et al. (2015) in Egyptian buffalo bulls, who found that the heritability estimates were 0.30, 0.38, and 0.35 for VOL, LS, and MM, respectively. In dairy cattle, Mathevon et al. (1998) investigated semen traits in Canadian Holstein Friesian bulls and found that the estimates of heritability were 0.24 and 0.31 for VOL and MM, respectively, in young bulls. Similarly, Druet et al. (2009) reported heritability estimates of 0.22, 0.43, and 0.19 for VOL, MM, and CONC, respectively, in Holstein Friesian bulls. Furthermore, Yin et al. [3] reported heritability estimates of 0.15, 0.12, and 0.22 for VOL, MM, and CONC, respectively, in Chinese Holstein Bulls. In Egypt, El-Komey et al. (2016) reported heritability estimates of 0.32, 0.14, and 0.18 for VOL, CONC, and LS, respectively, in Friesian bulls. Khattab et al. (2022) also evaluated some semen traits of Friesian bulls and reported heritability estimates of 0.31, 0.32, 0.29, and 0.33 for VOL, MM, CONC, and LS, respectively, and repeatability estimates of 0.47, 0.43, 0.56, and 0.44 for the same traits, respectively. The differences in heritability estimates reported in different studies can be due to differences in the genetic variation of the examined animals, age and maturity of bulls, method of estimation of the variance components, environmental deviations, and large standard errors due to small datasets.

**Table 3:** Estimates of variance components and genetic parameters (standard error) for semen traits (total number of records is 7761) in Egyptian buffalo bulls

Parameter	Ejaculate volume (mL)	Mass motility (%)	Live sperm (%)	Abnormal sperm (%)	Concentration^†^
*σ*^2^_*a*_	0.30435	241.28	200.23	0.11976	0.024
*σ*^2^_*PE*_	0.20449	134.83	106.97	0.079	0.013
*σ*^2^_*d*_	0.007	0.021	0.02	0.009	0.00004
*σ*^2^_*e*_	2.8342	59.039	57.935	2.93	0.008
*σ*^2^_*p*_	3.3504	435.17	365.15	3.1306	0.046
*d*^2^	0.002 (0.002)	0.006 (0.007)	0.006 (0.008)	0.003 (0.003)	0.001 (0.001)
*c*^2^	0.057 (0.06)	0.299 (0.26)	0.29 (0.24)	0.024 (0.027)	0.289 (0.239)
*h*^2^	0.08 (0.07)	0.52 (0.26)	0.51 (0.25)	0.04 (0.039)	0.49 (0.245)
*r*	0.14 (0.08)	0.82 (0.08)	0.79 (0.09)	0.06 (0.04)	0.78 (0.097)

^†^Sperm concentration (10^9^ spermatozoa/mL); *σ*^2^_*a*_ = additive genetic variance; *σ*^2^_*PE*_ = permanent environmental variance; *σ*^2^_*d*_ = variance due to test-day; *σ*^2^_*e*_ = residual variance; *σ*^2^_*p*_ = total phenotypic variance; *d*^2^ = proportion of phenotypic variance due to test-day effects; *c*^2^ = proportion of phenotypic variance due to permanent environmental effects; *h*^2^ = heritability; *r* = repeatability

**Table 4:** Estimates of genetic correlation (below diagonal) and phenotypic correlation (above diagonal) and their standard deviations for semen traits (total number of records is 7761) in Egyptian buffalo bulls

	Ejaculate volume	Mass Motility	Live sperm	Abnormal Sperm	Concentration^†^
Ejaculate volume		0.15 (0.36)	0.19 (0.33)	0.43 (0.23)	0.55 (0.27)
Mass motility	0.17 (0.74)		0.99 (0.01)	0.59 (0.25)	0.95 (0.14)
Live sperm	0.22 (0.72)	0.99 (0.01)		0.48 (0.33)	0.92 (0.19)
Abnormal sperm	0.85 (0.34)	0.89 (0.29)	0.76 (0.51)		0.50 (0.28)
Concentration^†^	0.82 (0.38)	0.95 (0.14)	0.92 (0.20)	0.82 (0.42)	

^†^Sperm concentration (10^9^ spermatozoa/mL)

### Genetic and phenotypic correlations

The phenotypic and genetic correlations between any two traits illustrate how a change in one character affects the other. Knowledge of genetic and phenotypic correlations is essential for generating selection indices and the selection for multiple traits. The majority of semen traits examined in this study had desirable genetic correlations with each other. Estimates of the genetic correlation for VOL, MM, LS, AS, and CONC traits ranged from 0.17 to 0.99. However, estimates of the phenotypic correlation between these traits ranged from 0.15 to 0.99 (Table 4). However, the high standard deviations of the estimates of genetic and phenotypic correlations, especially for VOL trait, may be due to the number of records and/or the relatively high variability. The genetic correlations between MM and each of LS and CONC were highly significant and were 0.99 ± 0.01 and 0.95 ± 0.14, respectively. Furthermore, the genetic correlation between LS and CONC was highly significant (0.92 ± 0.20). According to the present estimates, selecting Egyptian bulls for sperm mass motility will improve live sperm percentage and sperm concentration, which is a desirable goal for buffalo breeders and AI centers, and will enhance fertility, which is one of the most relevant economic traits in dairy cattle and buffalo. These results were consistent with those presented by Khattab et al. (2015) who reported that the estimate of genetic correlation between MM and LS was 1.0 ± 0.03, implying that the two traits are genetically associated, and the selection of one of them will be accompanied by an improvement in the other. Barth and Waldner (2002) estimated a genetic correlation of 0.84 between MM and LS in beef cattle bulls. Moreover, Druet et al. (2009) reported a genetic correlation of 0.58 between MM and LS in Holstein bulls. El-Komey et al. (2016) reported a genetic correlation of 0.67 ± 0.10 between LS and CONC in Egyptian Friesian bulls. Furthermore, Khattab et al. (2022) reported genetic correlation estimates of 0.60 ± 0.04 and 0.64 ± 0.01 between MM and each of CONC and LS, respectively, and 0.68 ± 0.01 between CONC and LS in Egyptian Friesian bulls. Herein, the phenotypic correlations between MM and each of LS and CONC were 0.99 ± 0.01 and 0.95 ± 0.14, respectively, and that between LS and CONC was 0.92 ± 0.19. These favorable and highly significant phenotypic correlations between the three traits were in agreement with those observed by Khattab et al. (2015) who reported a phenotypic correlation of 0.92 between MM and LS in Egyptian buffalo bulls. Another study on Friesian dairy bulls reported phenotypic correlation estimates of 0.43 ± 0.57 and 0.67 ± 0.12 between MM and each of CONC and LS, respectively (2022).

In conclusion, the year of semen collection and age of bull at semen collection exerted significant effects on semen traits of Egyptian buffalo bulls. However, the season of semen collection did not exert relevant impact on the examined semen traits. The high heritability estimates for MM, LS, and CONC, as well as the positive high significant genetic correlation between these traits, suggested that direct selection for MM could be an efficient method to increase semen quality in Egyptian buffalo bulls and, thus, improve fertility.

## Data Availability

Available upon request.

## Code availability

Available upon request.
